# Subsurface Fracture Mapping in Adhesive Interfaces Using Terahertz Spectroscopy

**DOI:** 10.3390/ma19020388

**Published:** 2026-01-18

**Authors:** Mahavir Singh, Sushrut Karmarkar, Marco Herbsommer, Seongmin Yoon, Vikas Tomar

**Affiliations:** School of Aeronautics and Astronautics, Purdue University, West Lafayette, IN 47907, USA; sing1042@purdue.edu (M.S.); yoon164@purdue.edu (S.Y.)

**Keywords:** terahertz time domain spectroscopy, fracture toughness, epoxy, sub-surface crack detection

## Abstract

Adhesive fracture in layered structures is governed by subsurface crack evolution that cannot be accessed using surface-based diagnostics. Methods such as digital image correlation and optical spectroscopy measure surface deformation but implicitly assume a straight and uniform crack front, an assumption that becomes invalid for interfacial fracture with wide crack openings and asymmetric propagation. In this work, terahertz time-domain spectroscopy (THz-TDS) is combined with double-cantilever beam testing to directly map subsurface crack-front geometry in opaque adhesive joints. A strontium titanate-doped epoxy is used to enhance dielectric contrast. Multilayer refractive index extraction, pulse deconvolution, and diffusion-based image enhancement are employed to separate overlapping terahertz echoes and reconstruct two-dimensional delay maps of interfacial separation. The measured crack geometry is coupled with load–displacement data and augmented beam theory to compute spatially averaged stresses and energy release rates. The measurements resolve crack openings down to approximately 100 μm and reveal pronounced width-wise non-uniform crack advance and crack-front curvature during stable growth. These observations demonstrate that surface-based crack-length measurements can either underpredict or overpredict fracture toughness depending on the measurement location. Fracture toughness values derived from width-averaged subsurface crack fronts agree with J-integral estimates obtained from surface digital image correlation. Signal-to-noise limitations near the crack tip define the primary resolution limit. The results establish THz-TDS as a quantitative tool for subsurface fracture mechanics and provide a framework for physically representative toughness measurements in layered and bonded structures.

## 1. Introduction

Adhesively bonded joints enable lightweight structural design, but their fracture characterization remains fundamentally constrained by how crack length is measured. Mode I double-cantilever beam configurations are widely used because the energy release rate can be obtained from load, compliance, and crack length using closed-form or augmented beam formulations developed for bonded beams and adhesive layers [1,2,3,4]. These formulations implicitly assume that the crack front used in the analysis represents the true interfacial separation state. In practice, however, crack fronts in bonded specimens can be curved and non-uniform across the width, particularly near free edges where three-dimensional constraint and bending fields perturb the local opening response [5,6]. Any crack length inferred from a single surface trace can therefore bias fracture parameters, especially when the opening is wide and the subsurface front deviates from the edge-visible path.

Bisphenol-A diglycidyl ether (BADGE)-based, amine-cured two-part epoxy adhesives are among the most widely used classes of structural adhesives due to their stable curing behavior, well-characterized mechanical response, and broad applicability in bonded joints [7]. When studied as thin adhesive layers using terahertz time-domain spectroscopy (THz-TDS), particularly in assemblies involving thicker adherends, enhancement of the dielectric response becomes critical. This can be systematically achieved by adding high-permittivity ceramic fillers such as strontium titanate (SrTiO_3_), which increase the effective dielectric permittivity and improve sensitivity to internal deformation and interfacial changes without altering the polymer network [8,9]. Transmission-mode measurements further constrain the choice of adherend materials, as commonly used metallic substrates exhibit strong reflectivity in this frequency range and inhibit through-thickness interrogation [10]. High-density polyethylene (HDPE) provides favorable transmission characteristics and is widely reported as a low-loss polymer in this regime, enabling millimeter-scale penetration while maintaining a mechanically representative bonded-joint configuration [11].

Surface-based experimental methods improve the richness of fracture data [12], but they do not remove the observability limitation when the interface is buried in an optically opaque substrate. Digital image correlation (DIC) can extract full-field surface kinematics for fracture assessment [13,14,15], yet it still measures a surface response and not the subsurface front location. The same limitation applies to optical crack tracing whenever the front is not co-planar with the observed surface. In bonded polymer systems, this limitation is amplified because the adhesive process zone and large crack opening can coexist with non-uniform width-wise advance, making edge tracking particularly sensitive to where the measurement is taken [5,6]. As a result, a mechanics-consistent crack length requires volumetric or subsurface access to the separation morphology rather than an edge-only surrogate. The optical spectroscopic methods have been used to access intrinsic response of materials at molecular level, including pressure, temperature, and energy dissipation [16,17].

Thz-TDS provides a complementary route because the transmitted or reflected terahertz waveform encodes internal optical path length variations that arise from dielectric contrast and internal discontinuities. Foundational time-domain THz work established broadband generation and detection [18,19,20], as well as frequency-domain extraction of complex optical constants and thickness from time-domain signals [21,22,23,24]. These capabilities have matured into practical multilayer thickness determination workflows, including for unknown materials and layered stacks [25]. Recent work has demonstrated accurate thickness and layer characterization of multilayer polymer coatings using THz-TDS, even in systems containing scattering fillers and metallic inclusions [26]. Terahertz imaging has accordingly been applied to subsurface inspection problems where optical methods fail, including three-dimensional imaging of opaque materials [27], impact and damage assessment in fiber-reinforced composites [28], defect mapping in building materials [29], and consolidation or layer separation in cultural heritage laminates [30]. Recent studies also demonstrate terahertz sensitivity to crack-like defects in engineered coatings, enabling detection and geometric interpretation when dielectric contrast is adequate [31]. These application studies collectively establish that terahertz imaging can resolve buried discontinuities at sub-millimeter scales in nonconducting systems.

A second, equally important requirement for quantitative fracture is that signal processing must reliably separate overlapping echoes [32] and stabilize the time-delay estimation that underpins geometric reconstruction. Advanced parameter-estimation strategies, including maximum-likelihood formulations, have been shown to significantly improve robustness of optical constant and thickness extraction in noisy THz-TDS data, particularly in multilayer systems with overlapping echoes [33]. Echo separation and parameter estimation in THz-TDS have been advanced through transfer function and optimization-based approaches [23,34], and through algorithms that improve complex refractive index extraction in dispersive media and multilayer settings [35]. In parallel, terahertz system performance limits have been quantified through dynamic range and signal-to-noise methodologies, which define the detectability window for small optical path changes and thin gaps [36,37].

Feature extraction and classification approaches have been applied to THz pulsed imaging data to distinguish defect morphologies in protective coatings beyond simple threshold-based detection [38]. For imaging, deconvolution and variation-regularized reconstruction methods have been shown to improve interpretability of THz pulse data in heterogeneous materials by suppressing ringing and mitigating overlap artifacts that otherwise confound thickness and interface localization [39]. In contrast to optical or thermal imaging techniques that natively provide full-field, multi-pixel measurements [15], spectroscopic diagnostics such as Raman and THz-TDS are inherently point-based measurements, requiring either multi-beam or multi-fiber implementations [17], or raster scanning [8,9], to reconstruct spatially resolved maps. THz has also been employed for quantitative subsurface porosity mapping in geologic materials, illustrating the sensitivity of THz delay and attenuation to distributed internal voids rather than discrete cracks [40].

Despite these advances, most THz nondestructive evaluation studies stop at qualitative detection or thickness mapping and do not translate subsurface geometry into fracture-mechanics quantities. Conversely, fracture-mechanics analyses for bonded joints remain limited by the fidelity of the crack length input when the front is buried and non-uniform. This work aims to close that gap by integrating THz-TDS with displacement-controlled double-cantilever fracture tests on opaque adherends bonded by a dielectric-contrast-enhanced adhesive. Subsurface time-delay maps are processed to reconstruct the evolving crack-front morphology and to compute a width-averaged crack length that is consistent with the three-dimensional separation state. The reconstructed geometry is then coupled to established DCB energy-release-rate models for adhesive joints to obtain fracture toughness while explicitly addressing edge effects and front nonuniformity. By doing so, the study advances terahertz inspection from defect visualization to quantitative subsurface fracture mechanics in bonded polymer systems.

## 2. Materials and Methods

### 2.1. Sample Preparation

Mode-I interfacial fracture specimens were prepared in a double-cantilever beam (DCB) configuration using high-density polyethylene (HDPE) adherends bonded with a two-part structural epoxy adhesive (Araldite 2011, Huntsman, The Woodlands, TX, USA). The DCB geometry was selected as per ASTM D5528/D5528M-21 [41] to impose controlled mode-I loading at the adhesive interface, while the adhesive formulation was modified independently to enhance dielectric contrast for THz detection of the crack-tip profile.

**Figure 1:**
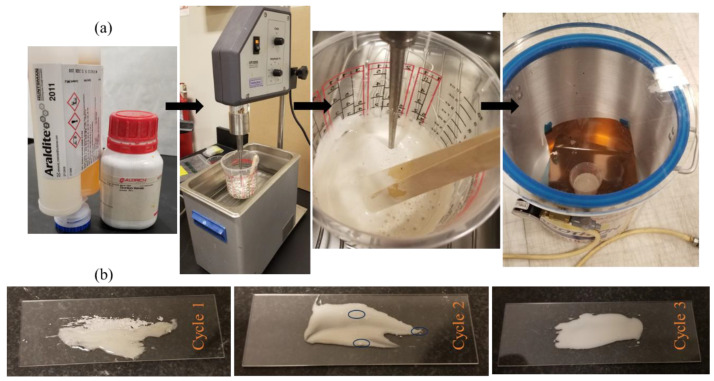
(**a**) Images illustrating key stages in the preparation of the Araldite–SrTiO_3_ (STO) adhesive system: mixing of the epoxy and STO particles, bubble evolution during sonication, and subsequent degassing under vacuum prior to specimen fabrication. (**b**) Optical images document the three-cycle mixing protocol: pronounced particle agglomeration after cycle 1, reduced but residual clustering after cycle 2 (highlighted by circles), and visually uniform dispersion with no observable clumping after cycle 3.

Strontium titanate (SrTiO_3_, STO) microparticles (Sigma-Aldrich, St. Louis, MO, USA) were added to the epoxy resin at 8 wt.%, corresponding to an estimated volume fraction of approximately 1.9%. The STO particles were dispersed into the resin using probe-assisted ultrasonication in a temperature-controlled water bath as shown in Figure 1a. Sonication was performed in three cycles of 10 min, each followed by a 5 min cooling interval to limit thermal rise and suppress premature curing. The bath temperature was continuously monitored using the integrated temperature sensor of the sonicator and maintained below 30 °C throughout mixing. Dispersion uniformity was assessed by glass-slide smear inspection, confirming the absence of visible particle agglomeration at the inspection scale. After particle dispersion, the hardener was added and mixed manually under low shear until the adhesive was visually homogeneous. The filled adhesive was then vacuum degassed (5 min at −25 in Hg pressure) to remove entrapped air prior to bonding as shown in Figure 1b.

**Figure 2:**
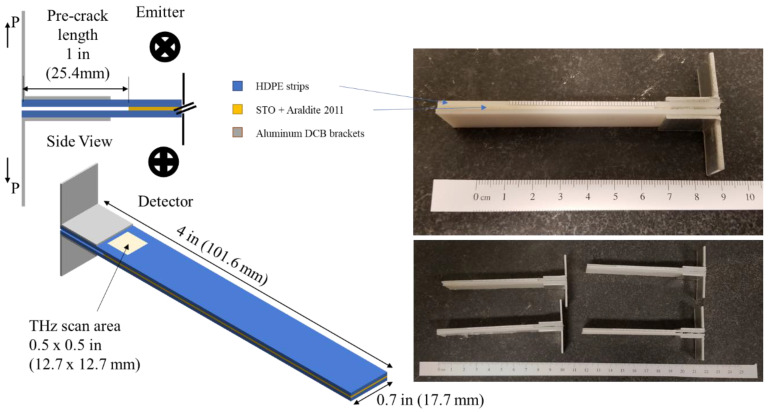
Final bonded specimen and terahertz scan geometry. Schematic of the double-cantilever beam specimen showing HDPE adherends, adhesive layer, initial pre-crack, loading direction, and the terahertz scan region used during fracture testing. Images of the fully assembled and cured specimen correspond to the schematic.

HDPE adherends were machined to final dimensions of 101.6 mm × 17.8 mm × 4.76 mm (Figure 2). The bonding surfaces were mechanically polished using 2500 grit paper and cleaned with isopropyl alcohol immediately before adhesive application. The STO-modified adhesive was applied to achieve a nominal interface thickness of 1.6 mm. A polytetrafluoroethylene (PTFE) insert was introduced at one end of the interface to define an initial pre-crack length of 25.4 mm. The bonded assemblies were cured at 40 °C for 24 h following the manufacturer-recommended cure schedule. After curing, “L”-shaped aluminum loading angles were bonded to the adherend ends using a polyurethane adhesive to facilitate load application during DCB testing. Prior to testing, the starter crack location and alignment were verified at the outer surface using optical microscopy.

### 2.2. Experiments

#### 2.2.1. Experimental Setup

**Figure 3:**
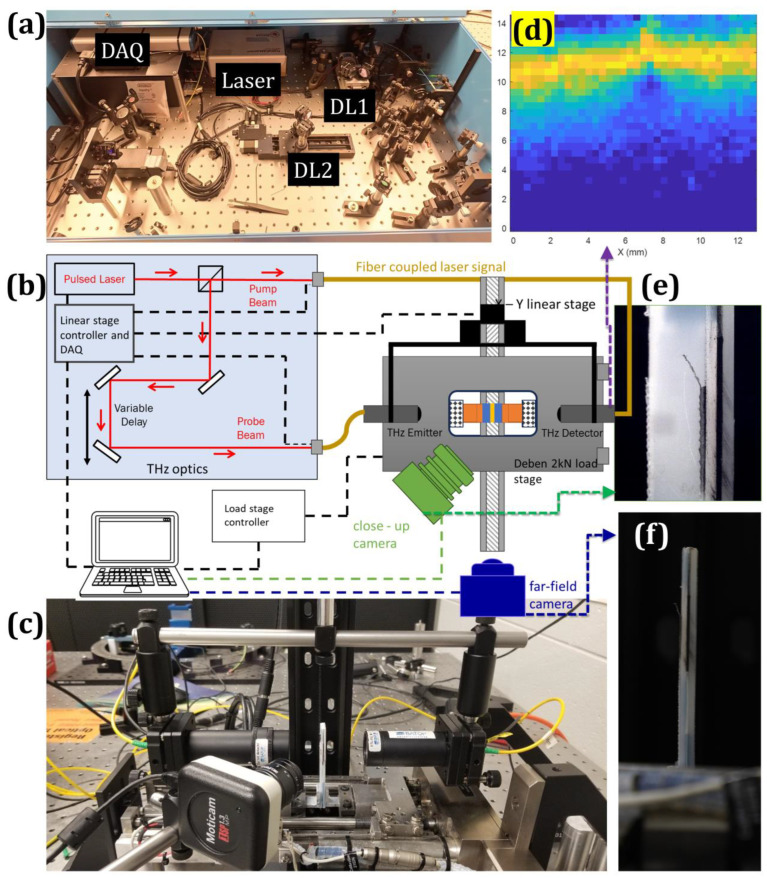
Integrated terahertz–fracture experimental configuration and representative measurements. (**a**) Terahertz time-domain spectroscopy system showing the femtosecond laser source, delay stage, data acquisition unit, and optical components. (**b**) Schematic of the terahertz beam path and experimental layout illustrating emitter–detector configuration, and integration with the double-cantilever beam loading stage, including near-field and far-field optical cameras. (**c**) Image of the loading stage with mounted terahertz optics and optical imaging systems. (**d**) Spatial map of the THz-TDS signal acquired over the same region. (**e**) Near-field optical image of the specimen around the initial crack region. (**f**) Far-field optical image capturing the full specimen geometry during testing.

Terahertz measurements were performed using a fiber-coupled terahertz time-domain spectroscopy (THz-TDS) system (TDS-1008-FO, Batop GmbH, Jena, Germany) operated in transmission geometry as shown in Figure 3a–c. The system employs photoconductive antenna (PCA) emitter–detector pairs driven by a femtosecond laser with a central wavelength of 785 nm. Optical excitation of the biased emitter generates a broadband terahertz pulse, while the detector samples. The transmitted electric field through optical pump–probe delay control, yielding the time-resolved waveform E(t).

The terahertz radiation was coupled into free space using silicon hyper-hemispherical lenses and guided through off-axis parabolic mirrors to form a collimated beam. At the specimen plane, 35 mm focal-length lenses mounted on both the emitter and detector antennas focused the beam, with a source–detector separation of 70 mm. This optical configuration produced a nominal terahertz spot size of approximately 0.4 mm at the adhesive layer. The beam was aligned nominally normal to the interface and propagated through the full thickness of the bonded assembly in transmission.

Mode-I fracture loading was applied using a Deben (Woolpit, UK) micro-tensile stage equipped with a 2 kN load cell. The double-cantilever beam specimen was mounted in a vertical peel-type configuration to enable symmetric opening of the adherends while maintaining unobstructed access for the terahertz transmission path. The mechanical loading axis and the terahertz propagation axis were arranged parallel and spatially offset by 25.4 mm, corresponding to the starter crack length. This offset geometry positioned the terahertz beam within the fracture region while avoiding obstruction from grips or loading hardware and ensured a constant beam location relative to the load line.

#### 2.2.2. Measurements

Mode-I fracture experiments were conducted under displacement control at a constant crosshead rate of 0.03 mm min^−1^. Load and crosshead displacement were continuously recorded throughout each test. Terahertz measurements were acquired at discrete loading states. At each selected load level, transmission-mode terahertz raster scans were performed over a 12.7 mm × 12.7 mm region of interest spanning the starter crack tip and extending along the adhesive layer (Figure 3d). The scan step size was 0.4 mm in both in-plane directions, yielding a 32 × 32 spatial grid per scan. During raster scanning, the mechanical loading stage remained fixed to avoid perturbation of the fracture configuration. The terahertz emitter–detector pair, mounted on a common translation platform, was translated relative to the specimen to acquire waveforms at each spatial location at the prescribed load state.

At each spatial location, a single THz-TDS waveform was acquired within a temporal window of 30–65 ps, using a temporal step size of 0.02 ps and an integration time of 0.15 s. These acquisition parameters were held constant across all scans. All measurements were performed at room temperature in ambient atmosphere under controlled laboratory humidity. For each specimen, a reference terahertz waveform was recorded in the same atmospheric conditions prior to mounting the sample. These reference measurements were used to normalize the transmitted waveforms acquired during fracture testing. The primary terahertz observable used for subsurface crack mapping was the time-domain peak position, which reflects local optical path length changes associated with interfacial separation and crack opening. Peak positions were extracted using a Fourier-based fitting routine implemented in the Batop T3DS (v4.0) software, and spatial maps were constructed from the extracted peak-delay values.

The 0.4 mm scan step is comparable to the nominal terahertz spot size (~0.4 mm), resulting in near-contiguous sampling of the probed region. Accordingly, the lateral mapping resolution is governed primarily by the spot size and signal-to-noise ratio rather than by the scan step. Interpolation was applied to the gridded data to reduce discretization artifacts and generate continuous contour fields suitable for crack-front delineation. To enhance crack-front contrast and suppress high-frequency artifacts, terahertz maps were further processed using an anisotropic diffusion-based image enhancement approach incorporating subpixel refinement and iterative unsharp masking. Alternative filtering strategies were evaluated but were not retained due to inferior crack-front clarity for the present dataset. To determine the crack position, the THz measurements were supported by optical imaging performed in near-field and far-field configurations to capture high spatial resolution (Figure 3e) and a larger field of view (Figure 3f), respectively.

### 2.3. Subsurface Crack-Front Reconstruction and Fracture-Parameter Estimation

Transmission-mode terahertz measurements provide spatial maps of the time-domain peak position of the transmitted electric field. The peak delay, denoted as τ(x,y), reflects local optical path length variations within the bonded assembly. As fracture progresses, the formation and growth of an air gap at the adhesive interface introduces a strong dielectric contrast, producing a measurable increase in the local peak delay. Accordingly, spatial variations in τ(x,y) provide a direct signature of subsurface crack opening and propagation within the adhesive layer.

For each loading state, two-dimensional peak-delay maps were constructed over the region of interest described in Section 2.2.2. The subsurface crack front was identified as the locus of maximum spatial gradient in the peak-delay field along the loading direction. The crack-front position was therefore defined implicitly by

(1) ∣∇τ(x,y)∣ → max,

where ∇τ denotes the in-plane gradient of the peak-delay field. This definition does not assume a straight or surface-parallel crack front and captures subsurface variations in crack advance within the adhesive layer.

An effective crack length was defined from the reconstructed subsurface crack front to enable fracture-mechanics analysis. For each load state, the effective crack length $a_{eff}$ was computed as the mean position of the reconstructed crack front along the specimen width

(2) $$\begin{array}{cccc} & a_{eff}=\frac{1}{W}\int_{0}^{W}a(y)\;dy, & & \end{array}$$

where a(y) is the local crack-front position and W is the specimen width. This definition preserves the influence of crack-front curvature while enabling the use of one-dimensional double-cantilever beam (DCB) relations.

Fracture parameters were evaluated using standard DCB mechanics formulations with the terahertz-derived effective crack length. The strain energy release rate G was calculated from the applied load P, specimen geometry, and compliance using

(3) $$\begin{array}{cccc} & G=\frac{P^{2}}{2B}\;\frac{dC}{da}{|}_{a=a_{eff}\;}, & & \end{array}$$

where B is the specimen width and C=δ/P is the compliance obtained from the measured load–displacement response. The derivative dC/da was evaluated using the effective crack length $a_{eff}$ rather than the surface-observed crack length.

By combining mechanically measured load–displacement data with terahertz-derived subsurface crack geometry, fracture parameters were obtained without relying solely on surface crack measurements. This framework explicitly incorporates subsurface crack evolution within the adhesive layer into the fracture analysis. The analysis assumes linear elastic behavior of the adherends and small-scale yielding within the adhesive layer under the applied loading conditions. Within these bounds, the reconstructed subsurface crack front provides a physically consistent basis for quantifying fracture evolution in bonded interfaces.

## 3. Results

### 3.1. Terahertz Waveform Signatures of Subsurface Interfacial Crack

**Figure 4:**
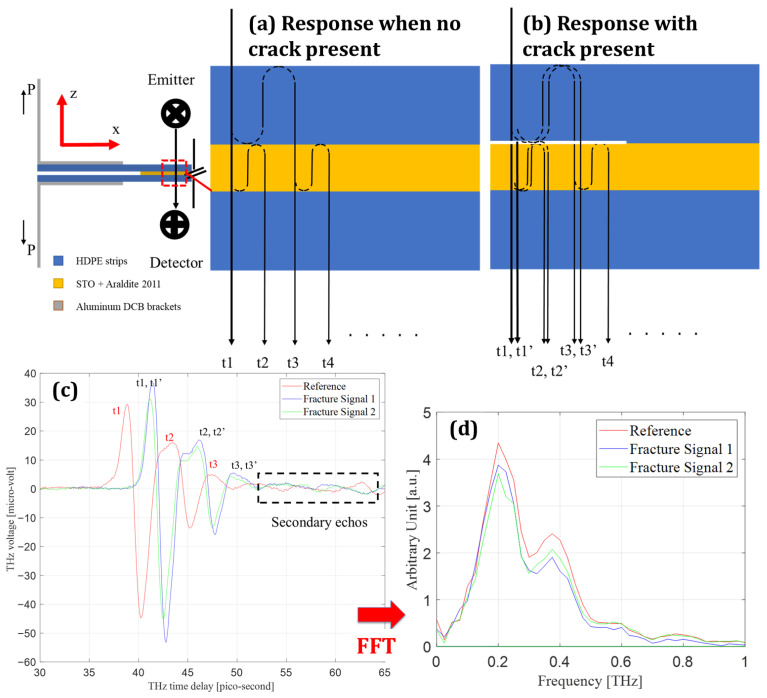
Terahertz wave interaction with an intact and separated adhesive interface. (**a**) Schematic of the intact HDPE–Araldite + STO interface illustrating terahertz transmission and reflections at successive material boundaries. (**b**) Schematic of the same interface after crack formation, showing the introduction of an air gap and the resulting additional optical path and reflection events. (**c**) Time-domain terahertz signals showing the reference waveform, a fracture signal acquired at a bonded location, and a fracture signal acquired at a separated location; labeled delay times correspond to the reflection paths indicated in (**a**,**b**). (**d**) Frequency-domain spectra obtained from the waveforms in (**c**), highlighting changes associated with interface separation.

Figure 4 presents the THz time- and frequency-domain signatures associated with subsurface interfacial cracking in the HDPE–adhesive–HDPE configuration. The formation of a crack introduces an internal air interface with a large refractive index contrast relative to the surrounding polymer layers, fundamentally altering wave propagation through the multilayer stack. Consequently, crack formation is manifested primarily through changes in phase accumulation and internal reflection pathways rather than through uniform attenuation of signal amplitude.

For a dielectric slab under normal incidence, the transmitted terahertz field is described as the coherent superposition of all transmission paths arising from Fresnel reflections at each interface. Following the multilayer formulation adopted in this work, the transmitted spectrum is expressed as

(4) $$\begin{array}{c} E_{t}\left(\omega \right)=\tau \tau^{\prime }exp\left[-j\;{\hat{n}}_{s}\left(\omega \right)\frac{\omega l}{c}\right][1+\rho^{\prime 2}exp\left(-2j\;{\hat{n}}_{s}\left(\omega \right)\frac{\omega l}{c}\right)\\ +\rho^{\prime 4}exp\left(-4j\;{\hat{n}}_{s}\left(\omega \right)\frac{\omega l}{c}\right)+\cdots \;]E^{0}\left(\omega \right), \end{array}$$

which may be written compactly as

(5) $$E_{t}\left(\omega \right)=\tau \tau^{\prime }exp\left[-j\;{\hat{n}}_{s}\left(\omega \right)\frac{\omega l}{c}\right]FP\left(\omega \right)\;E^{0}\left(\omega \right),$$

where ${\hat{n}}_{s}(\omega )=n_{s}(\omega )-j\kappa_{s}(\omega )$ is the complex refractive index of the layer, l is the effective propagation thickness, and FP(ω) is the Fabry–Pérot recirculation term accounting for multiple internal reflections. Introduction of a crack modifies both the number of admissible internal paths and the accumulated phase associated with each path, thereby perturbing the interference structure embedded in FP(ω).

The experimentally measured quantity is the reference-normalized transfer function $H(\omega )=E_{sample}(\omega )/E_{ref}(\omega )$, which for the present configuration is written as

(6) $$H\left(\omega \right)=\frac{4n_{s}\left(\omega \right)}{{\left[n_{s}\left(\omega \right)+1\right]}^{2}}\cdot \exp\left\{-\kappa_{s}\left(\omega \right)\frac{\omega l}{c}\right\}\cdot \exp\left\{-j\left[n_{s}\left(\omega \right)-1\right]\frac{\omega l}{c}\right\}$$

The optical constants used to interpret crack-induced phase variations are obtained from

(7) $$n_{s}\left(\omega \right)=1-\frac{c}{\omega l}∠H\left(\omega \right)$$

(8) $$\kappa_{s}\left(\omega \right)=\frac{c}{\omega l}\left\{\ln\left[\frac{4n_{s}\left(\omega \right)}{{\left(n_{s}\left(\omega \right)+1\right)}^{2}}\right]-\ln\left|H\left(\omega \right)\right|\right\}$$

Equations (6)–(8) establish that phase delay is the primary crack-sensitive observable, while amplitude variations play a secondary role through attenuation and interference effects.

Figure 4c compares representative time-domain waveforms acquired over intact and cracked regions. The cracked waveform exhibits a systematic temporal delay of dominant extrema relative to the intact reference, accompanied by a redistribution of oscillatory amplitudes across successive lobes. Importantly, the broadband temporal envelope remains preserved, indicating that the observed changes originate from modified phase accumulation and internal reflections rather than from bulk absorption or scattering losses. Figure 4d shows the corresponding frequency-domain spectra, where both signals retain comparable bandwidths but differ in spectral amplitude over the low-frequency range that dominates phase sensitivity. The concurrence of time- and frequency-domain modifications confirms that crack formation perturbs the phase term in Equation (6), consistent with the insertion of an additional internal interface.

The physical origin of these waveform changes is clarified by the interface-path interpretation shown in Figure 4a,b. For an intact interface (Figure 4a), the measured waveform comprises a sequence of returns $\left(t_{1},t_{2},t_{3},\ldots \right)$ associated with successive internal reflections between the HDPE and adhesive layers. Upon crack opening (Figure 4b), an air gap is introduced, generating additional reflection paths and shifted returns $\left(t_{1}^{\prime },t_{2}^{\prime },t_{3}^{\prime },\ldots \right)$. These additional paths alter both the timing and relative amplitudes of the measured features, producing the structured delay signatures observed experimentally. The presence of these returns in the measured waveform demonstrates that crack detection is governed by interface creation rather than by monotonic signal loss.

A direct implication of this multilayer behavior is that naïve peak tracking or amplitude thresholding is insufficient for reliable crack identification. Overlapping reflections can shift apparent extrema and obscure weaker returns, particularly near the crack tip where separation distances are small. The interface-based interpretation established in Figure 4 therefore motivates the spatial crack-front reconstruction strategy developed in the following section, where waveform-level delay information is transformed into two-dimensional maps that distinguish bonded, stretched, and fully separated regions of the adhesive layer.

### 3.2. Subsurface Crack-Front Reconstruction and Evolution

#### 3.2.1. Crack-Front Reconstruction from Enhanced Terahertz Delay Maps

**Figure 5:**
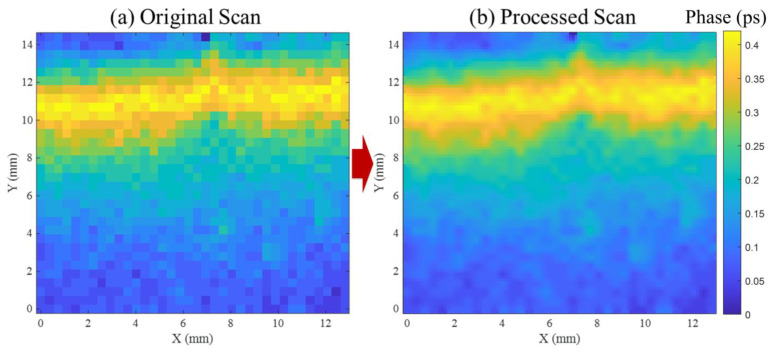
THz time-delay map processing for crack-front reconstruction. (**a**) Raw raster-scanned delay map showing pixel-level fluctuations from waveform noise and spatial sampling. (**b**) Enhanced map after anisotropic diffusion and unsharp masking, resolving the bonded–separated transition and enabling subsurface crack-front extraction.

Figure 5a shows a two-dimensional THz time-delay map acquired by raster scanning the bonded region at a fixed load-hold state. Each pixel represents the local time-of-flight shift extracted from the transmitted THz waveform and corresponds to the effective optical path length through the multilayer HDPE–adhesive–HDPE stack. The unprocessed map exhibits pixel-scale fluctuations within nominally uniform regions. Such spatial variability is expected in THz delay imaging of polymeric multilayers and adhesive bonds due to phase noise in the waveform, small thickness non-uniformities, and finite spatial sampling, and it can destabilize boundary identification if segmentation is performed directly on the raw map [42,43].

To enable stable crack-front reconstruction, the delay map was enhanced using an edge-preserving anisotropic diffusion update followed by unsharp masking. The nearest-neighbor directional differences used to compute local gradients are defined as

(9) $$\nabla_{a}I_{i,j}=I_{i-1,j}-I_{\left(i,j\right)},\;\;\nabla_{b}I_{i,j}=I_{i+1,j}-I_{\left(i,j\right)}$$

(10) $$\nabla_{c}I_{i,j}=I_{i,j+1}-I_{\left(i,j\right)},\;\;\nabla_{d}I_{i,j}=I_{i,j-1}-I_{\left(i,j\right)}$$

Here, $I_{i,j}$ denotes the delay value at pixel (i,j). Diffusion along each direction is controlled by conduction coefficients

(11) $$g_{a-d}=\frac{1}{1+{\left(\frac{\nabla_{a-d}I_{i,j}}{K}\right)}^{2}}\;,$$

where K is computed from local map statistics as

(12) $$K=2\times \left[\frac{mean\left(f_{i,j}\right)}{0.75\times \sigma \left(f_{i,j}\right)}\right]$$

The pixel values are iteratively updated in divergence form as

(13) $$I_{i,j}^{\prime }=I_{i,j}+0.25\left(g_{a}\nabla_{a}I_{i,j}+g_{b}\nabla_{b}I_{i,j}+g_{c}\nabla_{c}I_{i,j}+g_{d}\nabla_{d}I_{i,j}\right),$$

followed by unsharp masking to restore boundary sharpness and yield the final enhanced map $Q_{i,j}$. This processing route is used to suppress high-frequency, pixel-to-pixel fluctuations while preserving physically meaningful gradients, consistent with the purpose of anisotropic diffusion as an edge-preserving denoising operator [44]. The implementation used here follows the diffusion-based unsharp masking strategy described by Al-Ameen [45], which was selected after testing Gaussian smoothing, Laplacian filtering, and adaptive sharpening. Those alternatives either broadened the boundary or introduced spurious artifacts that complicate front extraction.

The enhanced map in Figure 5b resolves a spatially coherent delay field in which the boundary between lower-delay (bonded) and higher-delay (separated) regions becomes continuous and reproducible across the scan. The crack front is therefore extracted from $Q_{i,j}$ as a two-dimensional contour representing the separation boundary within the scanned area. Because the extracted front can vary across the specimen width, it is reduced to an effective crack length using the width-averaging definition introduced earlier in Equation (2), avoiding the implicit straight-front assumption associated with single-edge optical tracking.

#### 3.2.2. Evolution of Subsurface Crack Front Under Increasing Load

**Figure 6:**
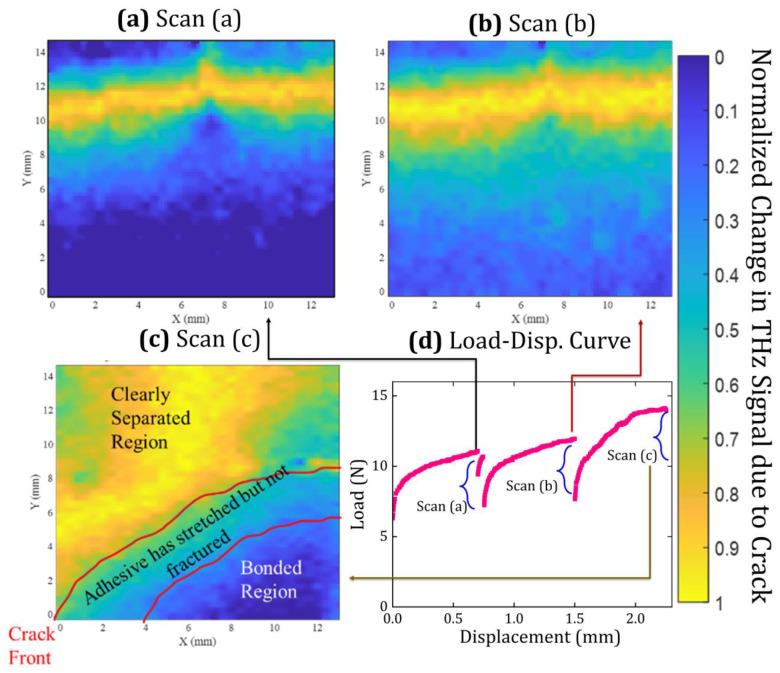
Subsurface crack evolution captured by THz time-delay mapping during displacement-controlled fracture. Normalized THz time-delay maps acquired at three displacement-hold states, corresponding to (**a**) scan A, (**b**) scan B, and (**c**) scan C, show the progressive opening and non-uniform advance of the buried interfacial crack. The corresponding mechanical response is shown in (**d**) as the load–crosshead displacement curve, with the three scan locations indicated. The sequence reveals a transition from a narrow, partially opened crack to a widened separation and finally to pronounced width-wise non-uniform crack propagation, demonstrating deviations from a planar crack front that are not accessible through surface-based measurements.

Figure 6 correlates the load–displacement response (Figure 6d) with subsurface crack-front maps (Figure 6a–c) acquired at three displacement-controlled load-hold states. Each map corresponds to a complete raster scan acquired while the crosshead displacement was held constant, providing a well-defined mechanical state for imaging. At the first load hold (Figure 6a), the delay map reveals a narrow-separated region and a crack front that is slightly inclined across the specimen width, with greater advance on the left side of the scanned region. This indicates that width-wise non-uniform advance can emerge early, even under nominally symmetric global loading.

At the second load hold (Figure 6b), the dominant change is widening of the separated region, while the forward advance of the crack-front contour is comparatively modest. The map therefore indicates an opening-dominated response in this regime, where increased separation behind the front contributes more strongly than front translation. Such opening-dominated behavior prior to significant crack growth is consistent with compliant polymer/adhesive interfaces and with the role of viscoelastic or rate-dependent deformation under load-hold conditions [46].

At the third load hold (Figure 6c), pronounced crack propagation is observed with substantial width-wise non-uniformity: the crack front advances significantly further on one side than the other, yielding a distinctly non-planar front within the mapped field of view. This is the key experimental consequence for fracture quantification. If crack length were inferred from a single specimen edge, measurement at the more advanced edge would overestimate the representative crack length, whereas measurement at the less advanced edge would underestimate it. Because the calculated fracture toughness depends strongly on the crack-length input in DCB-type reductions, either choice introduces systematic bias. Crack-front curvature and free-edge-driven morphology are reported in cantilever beam fracture experiments and are recognized sources of uncertainty when crack length is tracked at one edge and assumed uniform across the specimen width [47]. The terahertz-derived subsurface maps provide direct access to the crack-front morphology across the scanned width, enabling computation of a width-averaged crack length consistent with the actual interfacial separation state.

### 3.3. Edge Effects and Signal-to-Noise Constraints on Crack-Front Identification

The reconstruction of the subsurface crack front using THz-TDS relies on resolving small differences in time delay and phase associated with local changes in optical path length across the bonded interface. While fully separated regions introduce a strong and unambiguous delay signature due to the presence of an air gap, the region immediately ahead of the advancing crack front is characterized by small separations and partial interfacial opening. In this regime, edge effects associated with the transition from bonded to separated interfaces dominate the terahertz response, and the measured delay becomes increasingly sensitive to waveform noise, timing jitter, and phase uncertainty. These edge-effect-driven distortions directly limit the accuracy with which the crack-front position can be identified from terahertz measurements.

The measured terahertz signal in the frequency domain may be expressed as the sum of the true signal and a noise contribution

(14) $$S_{meas}(\omega )=S(\omega )+\Delta S_{n}(\omega ),$$

where $\Delta S_{n}(\omega )$ accounts for fluctuations arising from laser timing jitter, detector noise, and environmental perturbations. Under the common assumption that amplitude and phase noise are weakly correlated, this uncertainty propagates into the extracted optical constants and thickness. Following established treatments for THz-TDS uncertainty analysis, the uncertainty in the refractive index extracted from transmission measurements can be written as [36].

(15) $$\Delta n=\frac{\left(n-1+\frac{\omega }{c}n(n+1)d)\;n(1+n\right)}{|(n-1{)}^{2}+\left[(1+2n-n^{2})\kappa +n^{2}(1+n{)}^{2}\frac{\omega }{c}d\right]\frac{\omega }{c}d|}\frac{\Delta t}{t},$$

where n and κ are the refractive index and extinction coefficient, respectively, d is the effective thickness, and Δt/t represents the relative timing uncertainty. The corresponding uncertainty in thickness estimation for a given pulse order p is given by

(16) $$\Delta n_{p}\approx \frac{c}{\omega d^{2}}\frac{\phi_{p}}{2p+1}\Delta d=\left(\frac{1}{\left(2p+1\right)}-n\right)\frac{\Delta d}{d}$$

where $\phi_{p}$ denotes the phase of the p-th pulse. Subtracting thickness values obtained from adjacent pulses provides a direct estimate of the relative thickness error

(17) $$\frac{\Delta d}{d}\equiv SNR=\frac{\left(2p+1)(2q+1\right)}{2(q-p)}(n_{p}-n_{q}),$$

where p and q correspond to successive pulse indices. This formulation directly links the signal-to-noise ratio to the uncertainty in thickness estimation and, by extension, to the uncertainty in identifying the boundary between bonded and separated regions—particularly in edge-effect-dominated pixels near the crack front.

**Figure 7:**
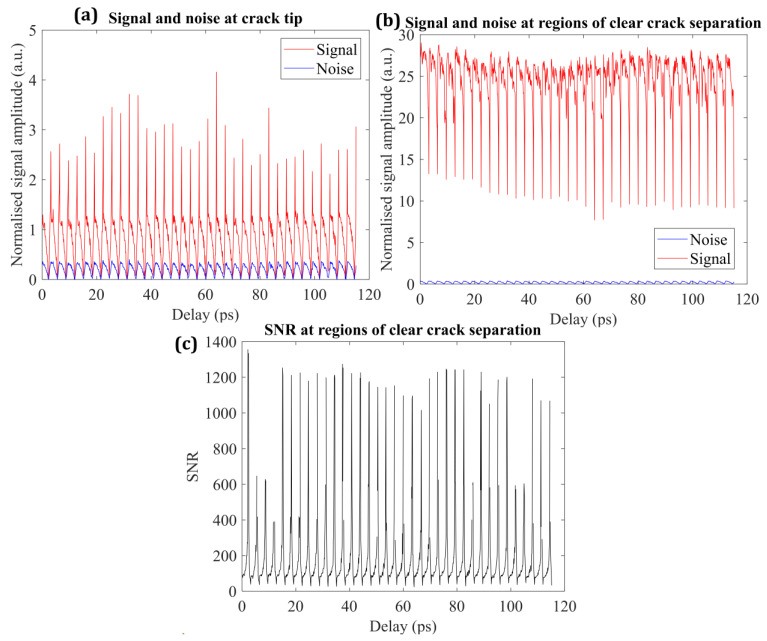
Terahertz signal and noise statistics during crack mapping: (**a**) signal intensity and noise level near the crack edge over a single scan, (**b**) signal and noise in a fully separated region away from the crack edge, and (**c**) signal-to-noise ratio as a function of time delay for the separated region. Lower signal-to-noise ratios near the crack edge limit reliable edge localization compared with regions of clear separation.

Figure 7 presents the experimentally evaluated signal, noise, and resulting signal-to-noise ratio for two representative regions: a fully separated crack region and the region immediately ahead of the crack front. In the fully separated region (Figure 7a), the measured signal amplitude remains high and stable, typically ranging from approximately 10 to 27 arbitrary units, while the noise level remains near baseline (below ~0.25 arbitrary units). The corresponding signal-to-noise ratio (Figure 7b) exceeds $10^{2}$ across most of the waveform window and reaches peak values on the order of $10^{3}$. In this regime, the propagated uncertainty in thickness is small compared to the measured separation, enabling reliable identification of the separated interface.

In contrast, the waveform statistics in the vicinity of the crack front (Figure 7c) show a pronounced reduction in signal amplitude, with values fluctuating near unity and intermittent spikes, while the noise magnitude remains comparable to that observed in the fully separated region. This behavior is consistent with edge effects, where overlapping echoes and mixed-phase sampling reduce waveform contrast and shift peak locations. As a result, the effective signal-to-noise ratio decreases substantially and becomes spatially variable. According to Equations (15)–(17), this reduction in SNR leads to a propagated thickness uncertainty that is comparable to the measured separation, limiting the precision with which the crack-front position can be inferred.

These observations establish a practical resolution limit for terahertz-based crack-front identification using the present THz-TDS configuration. Regions where the crack opening exceeds approximately 100 µm are mapped with high confidence, whereas the immediate crack-front neighborhood is intrinsically SNR-limited due to edge-effect-driven waveform overlap and reduced refractive index contrast. The reduced stability of crack-front contours near the leading edge observed in Figure 6 therefore arises from fundamental signal limitations rather than post-processing artifacts [32]. Similar signal-to-noise-driven constraints have been reported in terahertz-based thickness and defect measurements when the optical path difference approaches the system timing resolution [36].

### 3.4. Effective Stress Maps Inferred from Terahertz-Derived Fields

**Figure 8:**
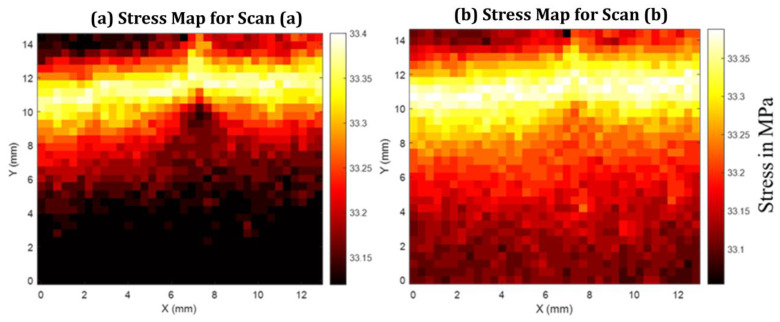
Average normal stress maps inferred from THz-TDS measurements for specimens containing a subsurface adhesive layer, corresponding to (**a**) scan point A and (**b**) scan point B on the load–displacement curve in Figure 6d.

Figure 8 presents spatial maps of the normal stress response reconstructed from terahertz-derived opening fields at two representative load-hold states corresponding to points a and b on the load–displacement curve in Figure 6d. The stress maps are obtained by converting the locally measured interfacial opening into an effective normal stress using the displacement-based elastic-foundation formulation introduced in Section 2.3.

The reconstruction is based on the local normal opening displacement δ(x,y) extracted from terahertz transmission measurements, which is related to the symmetric opening displacement $u_{y}(x,y)$ through $\delta =2u_{y}$. The effective normal stress is computed as

(18) $$\sigma_{z}=\frac{\left(1-\nu_{a}\right)E_{a}}{\left(1+\nu_{a}\right)\left(1-2\nu_{a}\right)+\nu^{2}\left(e^{-\frac{x}{3\nu_{a}t_{a}}}+e^{-\frac{y}{3\nu_{a}t_{a}}}\right)}\frac{2u_{y}}{t_{a}},$$

where $E_{a}$, $\nu_{a}$, and $t_{a}$ denote the elastic modulus, Poisson ratio, and thickness of the adhesive layer, respectively, and - x,y are the in-plane scan coordinates. This formulation follows generalized elastic-foundation models developed for adhesively bonded DCB specimens, which account for adhesive compliance and transverse constraint effects [3,4,6,48,49].

At the first load-hold state (Figure 8a), the reconstructed stress field is spatially smooth across the scanned region, with only weak variations observed near the subsurface crack front. Despite the presence of a clearly identifiable crack in the corresponding terahertz delay map, no sharp localization or singular stress feature is detected. At the second load-hold state (Figure 8b), the overall stress magnitude increases modestly while preserving a similar spatial distribution. The absence of pronounced stress gradients near the crack front is consistent across both loading states.

This behavior is physically consistent with the nature of the measurement and the specimen architecture. In transmission-mode THz-TDS, the measured phase delay integrates the optical path through the entire HDPE–adhesive–HDPE stack, and the resulting opening displacement represents a depth-averaged kinematic response rather than a layer-resolved adhesive deformation. For specimens with thin adhesive layers bonded to relatively thick polymer adherends, analytical and experimental studies have shown that the adherends dominate the global stiffness and load transfer, suppressing strong adhesive-layer stress signatures in averaged measurements [4,48,49]. Similar conclusions have been reported for elastic-foundation and beam-theory models of DCB joints, where local adhesive stresses contribute weakly to global response unless the adhesive thickness or stiffness is comparable to that of the adherends [1,3,50].

In fully separated regions, the terahertz response is governed primarily by the presence of an air gap, which introduces a large refractive index contrast and dominates the measured optical path length. In this regime, the terahertz signal is insensitive to mechanical stress transfer, and any stress values inferred from Equation (18) should be interpreted as cumulative indicators rather than stresses carried by the adhesive layer. Such dominance of geometric separation over stress-induced optical effects is intrinsic to transmission-mode terahertz measurements and has been noted in prior terahertz studies of multilayer systems, adhesive bonds, and subsurface defects [21,22,51].

### 3.5. Energy Release Rate Based on Subsurface Crack-Front Measurements

The mode-I energy release rate G was evaluated using Equation (3) defined in Section 2.3, with crack length obtained from the width-averaged subsurface crack front reconstructed from the terahertz delay maps. This definition is essential for the present specimen because the crack front exhibits measurable lateral non-uniformity; therefore, a single-edge crack-length measurement would not represent the interfacial separation state governing the global compliance and energy balance of the DCB configuration. The use of compliance and energy-based descriptions for DCB fracture, including formulations that account for adhesive compliance through elastic-foundation and augmented-beam models, is well established [3,4,6,48,49].

**Figure 9:**
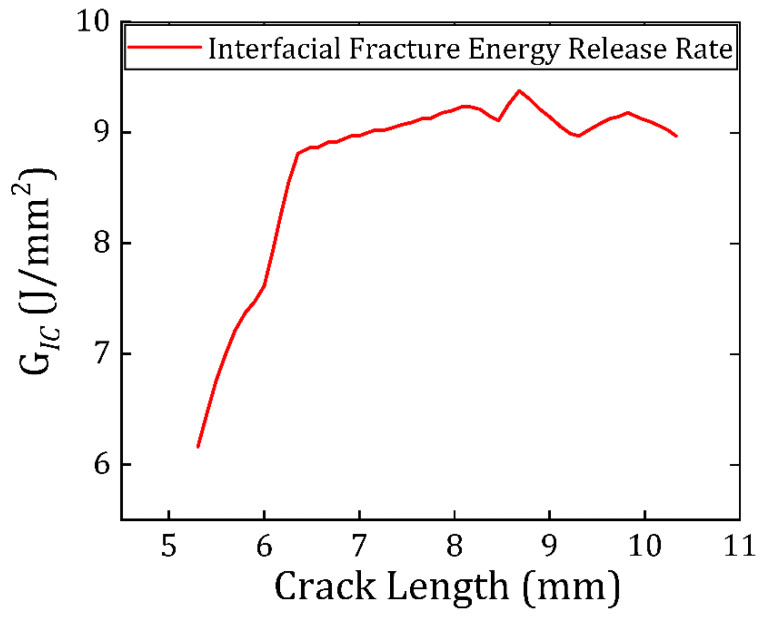
Mode I critical strain energy release rate for Araldite 2011 doped with STO particles bonded with HDPE substrate in DCB tests.

Figure 9 reports G as a function of the effective crack length a computed from the terahertz-derived crack-front reconstructions. The measured response shows a systematic evolution from an initial regime in which G varies with crack extension to a regime in which G approaches an approximately crack-length-insensitive level over the measured propagation window. In DCB fracture of adhesively bonded joints, such a transition is consistent with the development of a stable fracture process zone and the approach toward a steady propagation condition under displacement control, particularly when adhesive-layer deformation is non-negligible and must be represented through elastic-foundation or augmented DCB frameworks rather than by purely clamped-beam assumptions [3,4,6,48].

The principal contribution of the terahertz measurement to the G evaluation is the geometric fidelity of crack-length determination. As shown by the subsurface maps (Figure 6), crack advance is not uniform across the specimen width, producing a non-planar front. In this case, any edge-based crack tracking would return a crack length that is biased relative to the width-averaged value required by the global energy formulation, thereby biasing the inferred G. This sensitivity to geometric assumptions is inherent to DCB analysis, because crack length enters explicitly through compliance relations and energy-release-rate expressions derived from beam, foundation, or augmented-beam models [1,3,4,48]. The terahertz-derived width-averaged crack length reduces this bias by incorporating lateral variations in crack advance directly into the crack-length metric used in the fracture calculation.

The energy-release-rate calculation does not require the experiment to resolve a near-tip stress singularity within the adhesive layer; rather, it requires (i) mechanically consistent global loading data and (ii) a crack-length definition that reflects the actual interfacial separation geometry used by the DCB model. This separation between local-field resolution and global energy estimation is consistent with the analytical structure of DCB formulations, including augmented and elastic-foundation descriptions that incorporate adhesive compliance effects without requiring direct measurement of near-tip stresses [3,4,48,49].

### 3.6. Practical Limits and Pathways for Advancement

The present terahertz-based approach enables direct, non-destructive reconstruction of subsurface crack morphology during interfacial fracture; however, several practical constraints define its current resolution envelope. Raster-scanned THz-TDS imaging under displacement-controlled load-hold conditions imposes finite acquisition times, during which viscoelastic relaxation of the polymeric constituents may occur. While load stabilization protocols mitigate transient effects, the measured response represents a quasi-steady state rather than an instantaneous crack configuration. In addition, crack-front localization in the near crack-tip region is limited by signal-to-noise-driven edge effects, as small interfacial separations produce optical path differences comparable to the system timing uncertainty. This limitation is intrinsic to the measurement sensitivity and not a consequence of post-processing or segmentation methodology.

The terahertz delay maps further represent a depth-averaged optical response of the adhesive–adherend stack, such that the inferred stress distributions correspond to averaged stresses at each scan location rather than stresses confined to the adhesive layer alone. Given the relative thickness and stiffness of the HDPE adherends, their contribution dominates the measured response, which explains the absence of a pronounced stress singularity near the crack front. Despite these constraints, the method robustly captures crack-front curvature, width-wise non-uniformity, and crack opening evolution—features that are inaccessible to surface-based measurements and directly impact fracture-toughness evaluation. Future advances in detector dynamic range, reflection-mode sensitivity, and multiplexed or line-array acquisition are expected to extend spatial resolution toward the near crack-tip region while enabling faster, truly time-resolved fracture mapping.

## 4. Conclusions

This study establishes terahertz time-domain spectroscopy as a quantitative, non-destructive technique for resolving subsurface crack evolution during interfacial fracture. By integrating transmission-mode THz-TDS with physics-based signal processing and fracture-mechanics analysis, the work demonstrates direct experimental access to crack-front morphology, crack opening, and fracture-relevant fields in a double-cantilever beam configuration.

A central finding of this work is that surface-based crack-length measurements can be fundamentally unreliable in interfacial fracture experiments involving wider cracks. Subsurface terahertz delay maps reveal pronounced width-wise crack-front non-uniformity, with asymmetric crack advance across the specimen width under nominally symmetric loading. In such cases, crack-length measurements taken from a single specimen edge would systematically overestimate or underestimate the true crack advance, leading to biased fracture toughness values. Width-averaged crack lengths extracted from full subsurface crack-front reconstructions provide a more physically representative measure of interfacial separation.

Depth-averaged terahertz thickness maps were further used to compute spatial distributions of average stress and energy release rate under displacement-controlled loading. While the measured stresses represent cumulative contributions from the adhesive layer and thick adherends, their spatial evolution captures the mechanics governing crack opening and propagation. Signal-to-noise analysis shows that crack-front localization near the leading edge is intrinsically limited by timing uncertainty, whereas regions with crack opening approximately 100 μm are resolved with high confidence.

This work advances terahertz spectroscopy from defect visualization to a mechanics-informed fracture diagnostic. By enabling subsurface crack-front mapping and width-averaged fracture measurements, the approach provides capabilities that are not accessible with conventional surface-based methods and offers a foundation for future high-resolution and in situ interfacial fracture characterization.

Future work will focus on improving the temporal efficiency of terahertz scanning to minimize time-dependent relaxation effects during mechanical loading. This can be achieved by implementing multi-point acquisition strategies or reduced-dimensional line-scan approaches, enabling faster capture of spatially resolved delay fields. Such developments will facilitate measurements under effectively monotonic loading conditions and improve the fidelity of crack-opening and crack-front evolution data.

## Data Availability

The original contributions presented in this study are included in the article. Further inquiries can be directed to the corresponding author.

## Nomenclature

**Symbol**	**Description**	**Units**
a_eff_	Width-averaged effective crack length	m
a(y)	Local crack-front position along specimen width	m
B	DCB Specimen width	m
c	Speed of light in vacuum	m s^−1^
δ	Crack opening displacement	m
δ(x, y)	Local crack opening displacement	m
Eₐ	Elastic modulus of adhesive	Pa
E_ref(ω)	Reference spectrum	a.u.
E_sample(ω)	Sample spectrum	a.u.
E^0^(ω)	Reference electric-field spectrum	a.u.
Eₜ(ω)	Transmitted terahertz electric-field spectrum	a.u.
FP(ω)	Fabry–Pérot recirculation term	–
G	Mode-I strain energy release rate	J m^−2^
H(ω)	Transfer function	–
∠H(ω)	Phase of transfer function	rad
kₛ(ω)	Extinction coefficient	–
l	Effective propagation thickness	m
νₐ	Poisson’s ratio of adhesive	–
${\hat{n}}_{s}(\omega )$	Complex refractive index	–
nₛ(ω)	Real part of refractive index	–
ω	Angular frequency	rad s^−1^
P	Applied load	N
ρ’	Reflection coefficient	–
σ_z_	Effective normal stress	Pa
tₐ	Adhesive-layer thickness	m
τ, τ′	Transmission coefficients	–
τ(x, y)	Peak time-delay field mapped over the scanned area	s
∇τ(x, y)	In-plane gradient of the peak time-delay field	s m^−1^
uᵧ(x, y)	Symmetric crack opening displacement (δ = 2uᵧ)	m
W	Specimen width	m
